# Inaugural Address

**Published:** 1913-02

**Authors:** J. Edgar Paullin

**Affiliations:** Atlanta, Ga.


// Journal-Record of Medicine

Successor to Atlanta Medical and Surgical Journal, Established 1855
and Southern Medical Record, Established 1870
**OWNED BY THE ATLANTA MEDICAL JOURNAL COMPANY**
PUBLISHED MONTHLY
*Official Organ Fulton County Medical Society, State Examining Board, Presbyterian Hospital, Atlanta, Birmingham and Atlantic Railroad Surgeons' Association, Chattahoochee Valley Medical and Surgical Association, Etc.*

EDGAR BALLENGER, M. D., Editor
BERNARD WOLFF, M. D., Supervising Editor
A. W. STIRLING, M. D., C. M., D. P. H.; J. S. HURT, B. Ph., M.D.
GEO. M. NILES, M. D., W. J. LOVE, M. D., (Ala.); Associate Editors
E. W. ALLEN, Business Manager
**COLLABORATORS**
DR. W. F. WESTMORLAND, General Surgery
F. W. McRAE, M. D., Abdominal Surgery
H. F. HARRIS, M. D., Pathology and Bacteriology
E. B. BLOCK, M. D., Diseases of the Nervous System
MICHAEL HOKE, M. D., Orthopedic Surgery
CYRUS W. STRICKLER, M. D., Legal Medicine and Medical Legislation
E. C. DAVIS, A. B., M. D., Obstetrics
E. G. JONES, A. B., M. D., Gynecology
R. T. DORSEY, JR., B. S., M. D., Medicine
L. M. GAINES, A. B., M. D., Internal Medicine
GEO. C. MIZELL, M. D., Diseases of the Stomach and Intestines
L. B. CLARKE, M. D., Pediatrics
EDGAR PAULIN, M. D., Opsonic Medicine
THEODORE TOEPEL, M. D., Mechano Therapy
R. R. DALY, M. D., Medical Society
A. W. STIRLING, M. D., Etc., Diseases of the Eye, Ear, Nose and Throat
BERNARD WOLFF, M. D., Diseases of the Skin
E. G. BALLENGER, M. D., Diseases of the Genito-Urinary Organs

VOLUME LIX FEBRUARY, 1913 No. 11

### INAUGURAL ADDRESS

BY J. EDGAR PAULLIN, M. D., ATLANTA, GA.

A medical society well conducted should be a clearing house for ideas among its various members. We should occasionally stop and take sufficient time to look over our stock, run up the debit and and credit side of the ledger and see just where we stand. Being composed of human beings with human desires, we often fail to reflect over our errors and mistakes: How many men here assembled have read papers on such subjects as "The Mistakes I have made," or even better "On the Last One Hundred Mistakes I have made?" How many have heard papers on "Mistaken Diagnosis and Results of Poor Treatment?" From the lack of these subjects among our programmes is one to judge that no member knows sufficiently of these subjects to speak with authority, or is it that we hesitate to parade our short-comings before our fellow practitioners? Were we to have the annual stock-taking done thoroughly, just as we have the books of our society audited, much valuable information would be obtained. The practice of medicine at its very best is an exceedingly uncertain art based on probabilities. Variability is the law of life, no two faces are the same, no two bodies are alike, no disease manifesting itself the same in any two individuals; why then should we not take stock and gain from a valuable experience among ourselves morsels of truth that will benefit mankind?

It is the habit of many of us to run here and there and everywhere, to see the work of others, to gain new ideas, because every true physician seeks knowledge, and the greater number of them for knowledge's own sake. How few of us, on the other hand, know what our own neighbors are doing in our midst. Why don't we visit among ourselves more, and see what our friends and co-workers are accomplishing. Is it because we are afraid that our fellow doctor will casually remark, when he is in the society of the elect, "that Dr. So and So came around to-day and I showed him how to do such and such an operation, or make such and such a diagnosis?" Rivalry, jealousy, and the fear of having it generally known that we are seeing these things done by our brother doctor many times prevents this one great factor in the advancement of our profession. Firmly do I believe that just as good work is done by the members of this society as there is anywhere; so let us make it our duty to familiarize ourselves with it.

How many post-mortems are held upon cases where the diagnosis is obscure? How many of us ask permission of the family to perform an autopsy? Is this apparent timidity due to our fear of injuring the feelings of the relatives, or is it due to the fact that we are afraid our ante-mortem diagnosis will be upset, and that explanations will have to be made to the friends of our shortcomings? The post-mortem room is the most valuable asset we have to seek knowledge. Autopsies are obtained frequently with very little difficulty and never yet have I seen the relative's feelings wounded by a request for the operation. During the coming year it is to be hoped that the members will exhibit more of these specimens than have been shown in the past.

The exhibition of clinical cases and clinical specimens should be encouraged. Reports of cases with their diagnosis and treatments should be more often made. No more instructive work is possible than carefully and completely demonstrated specimens; specimens exhibited in the gross and the finer structures shown under the microscope. How valuable would it not be to have as a regular thing on the program for each meeting some doctor assigned to relate an unusual case with his diagnosis and treatment, or assign someone to exhibit a clinical case, or specimens.

With fear that the remark will be received with laughter because of our past upheavals and struggles on this subject, but realizing the importance, the paramount need by this society, and the absolute necessity to the individual members of a library, I gain courage to mention the matter in this presence. Of all the things that the members have accomplished toward the alleviation of suffering and relief from disease, when we should bow our heads in shame that this organization, the largest in the State, comprising at least one-sixth of the members of the State Medical Association, does not have the slightest evidence of a library for the members. Is it possible for us to arrange in some way to get ourselves stirred over the necessity of such an institution? Think of this society, the real god-father to two medical schools, with an enrollment of over five-hundred students without a library. How can the teachers in these schools expect to properly direct the thoughts of these young men without the assistance of a library? How can we expect our ranks to be yearly recruited with men who will find out things and enlighten us, if we do not place at their disposal the work of the master minds of medicine? How can we, gentlemen, expect to come to meetings of this society and hear the best papers when we do not place at the disposal of our members the stimulus necessary and essential for a good paper? Could we only feel the necessity of this great factor, the ways and means would soon be forthcomings, and Atlanta would have a medical library, second to none in the State. As it is the Atlanta Medical Library Association has reposing in the basement of the State Capitol a sufficient nucleus to begin its operation, and I would ask the society to appoint a committee to work out plans for this purpose. Our sister city, Savannah, has a library now, just three years old, with a wonderful collection of books, journals, etc. Can we not do equally as well?

Finally, is there not some way of increasing the attendance at our meetings? If there is a sensational report to be made by the Board of Censors, an election or other unusual excitement, we can easily get up a crowd—why then can't we have scientific papers with appropriate discussions sufficient to excite interest? The man who knows it all and who does not come to the society because he can get from "Dr. ——" text-book all the information in the paper to be read, is a rare quantity among our members, although we probably have some few; this man generally says that he feels better at home, and after all, probably it is the best place for him. The real reason is probably that many of them have become inoculated with that all too serious and all too fatal microbe "Apathy." Let us begin the year with a determination to overcome this infection by the establishment of sufficient antibodies to make us immune, to rid us of the pest, the mill-stone to progress.

With your help, individually and collectively it will be easy to make this society the best in the country, with the assurance of this, I as your president, will lend my every effort to serve you in the best possible way, working toward the goal which we all so desire to reach. *"Perfection."*

